# The immunology of traumatic brain injury: a prime target for Alzheimer’s disease prevention

**DOI:** 10.1186/1742-2094-9-185

**Published:** 2012-08-01

**Authors:** Brian Giunta, Demian Obregon, Renuka Velisetty, Paul R Sanberg, Cesar V Borlongan, Jun Tan

**Affiliations:** 1 James A. Haley Veterans’ Administration Hospital, 13000 Bruce B. Downs Blvd., Tampa, FL, 33612, USA; 2 Department of Psychiatry and Behavioral Neurosciences, Neuroimmunology Laboratory, Morsani College of Medicine, University of South Florida, 3515 E. Fletcher Ave., Tampa, FL, 33613, USA; 3 Department of Neurosurgery and Brain Repair, Center of Excellence for Aging and Brain Repair, Morsani College of Medicine, University of South Florida, 12901 Bruce. B. Downs. Blvd., Tampa, FL, 33612, USA; 4 Department of Psychiatry and Behavioral Neurosciences, Silver Child Development Center, Rashid Laboratory for Developmental Neurobiology, Morsani College of Medicine, University of South Florida, 3515 E. Fletcher, Tampa, FL, 33613, USA; 5 Department of Psychiatry and Behavioral Neurosciences, University of South Florida, Morsani College of Medicine, 3515 E. Fletcher Avenue, Tampa, FL, 33613, USA

**Keywords:** traumatic brain injury, flavonoids, microglia phenotype, Alzheimer’s disease, cytokines

## Abstract

A global health problem, traumatic brain injury (TBI) is especially prevalent in the current era of ongoing world military conflicts. Its pathological hallmark is one or more primary injury foci, followed by a spread to initially normal brain areas via cascades of inflammatory cytokines and chemokines resulting in an amplification of the original tissue injury by microglia and other central nervous system immune cells. In some cases this may predispose individuals to later development of Alzheimer’s disease (AD). The inflammatory-based progression of TBI has been shown to be active in humans for up to 17 years post TBI. Unfortunately, all neuroprotective drug trials have failed, and specific treatments remain less than efficacious. These poor results might be explained by too much of a scientific focus on neurons without addressing the functions of microglia in the brain, which are at the center of proinflammatory cytokine generation. To address this issue, we provide a survey of the TBI-related brain immunological mechanisms that may promote progression to AD. We discuss these immune and microglia-based inflammatory mechanisms involved in the progression of post-trauma brain damage to AD. Flavonoid-based strategies to oppose the antigen-presenting cell-like inflammatory phenotype of microglia will also be reviewed. The goal is to provide a rationale for investigations of inflammatory response following TBI which may represent a pathological link to AD. In the end, a better understanding of neuroinflammation could open therapeutic avenues for abrogation of secondary cell death and behavioral symptoms that may mediate the progression of TBI to later AD.

## Epidemiology of post-traumatic brain injury Alzheimer’s disease

It has been suggested that a long-term process of amyloid-beta (Aβ) metabolism is initiated by traumatic brain injury (TBI). Chronic axonal pathology seems to supply all of the needed machinery for both the anabolism and catabolism of Aβ [1]. These Aβ plaques formed in the initial weeks after injury may actually regress with time. In this case, a continuously renewed store of Aβ in degenerating axons can be kept in check through degradation by endogenous mediators such as neprilysin or adequate numbers of anti-inflammatory phagocytic microglia [1]. In a subset of TBI patients, the balance between Aβ anabolism and catabolism eventually shifts during the aging process, accounting for the epidemiologic evidence of a link between TBI and Alzheimer’s disease (AD) [1]. A deficiency in microglial clearance of Aβ could possibly account for this balance shift since aging microglia are known to have a reduced phagocytic capacity and this is observed in AD [2]; the most common age-related dementia. Indeed, in 2006 there were 26.6 million people with AD worldwide, and this number is predicted to quadruple by the year 2050 [3]. As such, an understanding of the mechanisms promoting AD risk is important. A history of TBI is a strong risk factor for AD [3-11], although there remains an incomplete consensus since some epidemiological studies have not uncovered such an association [12-18]. This is probably due to the retrospective nature of some studies that may have led to recall bias (systematic error due to inaccuracies in subjects’ ability to recall their history of TBI). This can be a confounding source especially when collecting data from patients regarding their cognitive impairments or from secondary informants [19]. Indeed, larger, more controlled studies, including level 1 evidence (which requires prospective examination and randomization) [20], have led to an overall acceptance that TBI is a risk factor for AD development [15].

Although TBI is typically believed to be a static pathological insult from a single event, new clinical unrecognized clinical symptoms can arise many years after the initial injury. Indeed, patients may display alterations in their daily living activities long after TBI and may develop AD [21].

Along these lines, there is evidence that a history of TBI accelerates the AD onset to younger ages [22-24] and that the more severe the injury, the greater the risk of developing AD [20,25]. Repetitive mild TBI especially promotes incapacitating consequences and AD-like cognitive deficits are reported in such cases. For example, in a study of 2,552 retired professional American football players there was a five-fold increase in the precursor to AD, mild cognitive impairment, and a threefold increase of reported significant memory problems among retirees with three or more reported concussions compared with retirees with no history of concussion [26]. This study also detected an earlier onset of AD in the retirees compared with the general American male population.

In other human studies, TBI has been shown to result in amyloid deposits reminiscent of AD pathology [27,28]. The first piece of evidence to suggest a mechanistic correlation between TBI and AD was the observation that Aβ plaques are found in up to 30% of patients who die acutely post TBI [27]. The senile plaques were found in all age groups, even children. Conversely, in control cases (patients who died from non-neurological causes), these plaques were detected almost exclusively in older individuals [27]. Additionally, plaques have even been observed in tissue surrounding contusions that was surgically removed from survivors of TBI [29]. Moreover, the neuritic plaques in TBI patients are very similar to those in the early stages of AD [19]. The major difference is that AD plaques develop slowly and are largely found in older people, whereas TBI-associated plaques can appear rapidly (within just a few hours) after injury [27]. The predominant form of Aβ in the plaques formed after TBI, and in the soluble Aβ found in the brains of these patients, is Aβ_42_ – which, as mentioned earlier, is prone to aggregate.

As TBI is a complex and heterogeneous syndrome, the type and severity of the acute inflammatory pathology probably has a central role in determining the risk of developing AD. Moreover, the baseline susceptibility or mental reserve of the patient may be predetermined by multiple factors including age, sex, and the interaction of several known or unknown genetic factors [19].

In the brains of patients suffering chronic TBI, several neuropathological hallmarks of AD (in addition to amyloid) have been noted – including neurofibrillary tangles, acetylcholine deficiency, and tau immunoreactivity [30]. A common feature of these pathologies is that they initiate and potentiate a brain inflammatory cascade that we hypothesize as the central mechanistic link between TBI and later development of AD.

## Inflammation links traumatic brain injury to later Alzheimer’s disease development

The initial inflammatory response of TBI [8,11,21] may be key to later AD development. This response results in neuronal injury and often in disruption of the blood–brain barrier. Microglial cells react to this injury within minutes, and stay activated chronically [31]. Once induced into this state, the microglia become nearly identical to peripheral macrophages, acting as antigen-presenting cells (APC) and secreting proinflammatory cytokines and chemokines [32,33]. For a full review of the activation states of microglia see Town and colleagues [34]. In animal models – including, but not limited to, fluid-percussion brain injury in rats [11], and combined unilateral lesion of the arm area of the primary motor cortex and arm area of the lateral premotor cortex in rhesus monkeys [21,35] – it was found that the initial inflammatory response persists for at least 1 year, especially in the thalamus [11,21,35].

In humans as well, postmortem studies have shown microglial activation many years after TBI [36]. Sites of activation often coincide with those of neuronal degeneration and axonal abnormality, and include disconnected nuclei such as the thalamus [8]. In humans, the positron emission tomography ligand ^3H^PK11195 detects subacute microglial activation at the site of strokes, as well as in remote white matter connected to the lesion [37,38]. Later, thalamic and brainstem microglial activation becomes evident due to the disconnection of these areas. Autoradiographic studies in rat models of TBI demonstrate an increased thalamic uptake of ^3H^PK11195 linked to a 31-fold increase in microglial numbers in the thalamus ipsilateral to a cortical injury. In a recent study of TBI patients using this modality, it was found that increased microglial activation can be present up to 17 years after TBI [36]. This observation indicates that TBI initiates a chronic inflammatory cycle and highlights the importance of considering the response to TBI as evolving over years or even decades [36]**.**

As stated earlier, many of the pathologies of TBI [39] are mediated through an inflammatory cascade characterized by activation of microglia [40,41] and through a concordant increase in proinflammatory cytokines [42,43]; both of which have the ability to exacerbate other pathologies including later dementia [44]. Microglia do not have simply one phenotypic manifestation [34]. As we have suggested previously, microglial cells exist in at least two functionally distinguishable states once activated – namely a phagocytic phenotype (innate activation) or the aforementioned antigen-presenting phenotype (adaptive activation) that is seen post TBI. When challenged with certain pathogen-associated molecular patterns (particularly CpG-DNA), murine microglia seem to activate a mixed response characterized by enhanced phagocytosis and proinflammatory cytokine production as well as adaptive activation of T cells. In the experimental autoimmune encephalitis model, murine microglia seem to largely support an adaptive activation of encephalitogenic T cells in the presence of the CD40–CD40 ligand interaction. In the context of Aβ challenge, CD40 ligation is able to shift activated microglia from innate to adaptive activation; similar to the scenario post TBI. Further, it seems that the cytokine milieu to which microglia are exposed biases these cells to adaptive activation (that is, anti-inflammatory Th2-associated cytokines such as IL-4, IL-10, and perhaps transforming growth factor-beta 1) or to an innate form of activation (that is, proinflammatory Th1-associated cytokines such as IFNγ, IL-6, and TNFα) [34]. In this regard, innate activation of microglia generally leads to amyloidogenic amyloid precursor protein (APP) processing and the generation of Aβ plaques [45-47]. Indeed, we have previously shown experimentally that blocking transforming growth factor-β–Smad2/3 innate immune signaling mitigates AD-like pathology [48].

Not all forms of microglial activation are deleterious, however, as activated microglia may serve a protective role in both TBI and AD. Regarding the former, at 3 hours post moderate fluid percussion in rats, the majority of new cells replacing the subventricular zone of the traumatized hemisphere were astrocytes, macrophage/microglia, oligodendrocytes, and neurons, with the majority of cells appearing glial in nature. These populations promoted neurogenesis in the granular cell layer of the hippocampus and suggest TBI stimulates widespread cellular proliferation for days after injury and results in focal neurogenesis in the dentate gyrus of the hippocampus promoted by microglia. These microglial responses to injury may therefore participate in brain repair and functional recovery [49].

Regarding the latter, it has been shown in Aβ_1–42_-immunized mouse models of AD that microglial phagocytosis of β-amyloid plaques is at least partly responsible for the therapeutic benefit in these animals [34]. Since inflammation post TBI can last for many years, and since this inflammation is promoted by APC-like microglia, if we could repolarize these cells to their phagocytic state before the inflammation becomes chronic it may be prophylactic for post-TBI AD. Moreover, this also suggests interventions to stop the conversion of TBI to AD may be beneficial for longer intervals after trauma than previously assumed [36].

## Intervention with natural flavonoids as a means of reducing the risk of Alzheimer’s disease after traumatic brain injury

The most common group of plant phenols, flavonoids are found in nuts, fruits, vegetables, grains, and tea. More than 6,000 different flavonoids have been described [50] and can be categorized into six classes by structure: flavonoles, flavones, isoflavones, flavanoles, flavanones, and anthocyanidins [51]. Flavonoids possess several physiological properties, including antioxidant, antibacterial, antiviral, antimutagenic, anticancer, and – most importantly – anti-inflammatory properties [52,53].

Flavonoids exist as glycoside and aglycone forms in plant-derived foods. After oral ingestion, flavonoids are extensively conjugated and metabolized during absorption in the small intestine and then again in the liver [54-56]. The intact form of flavonoid and the respective metabolites derived from flavonoid biotransformation in the gastrointestinal tract and in the liver are the forms that enter the circulation and ultimately reach the brain [57-59].

Several flavonoids have shown potential for the treatment of TBI symptoms in humans and animals. In this regard, Diamond and colleagues reviewed all researched conference proceedings and research papers identified in Medline, in the Research Council for Complementary Medicine database based on the British Library database, and in PsychInfo [60]. The review was extensive in that controlled clinical studies with both positive and negative findings were included, in addition to animal studies providing mechanisms of activity. The following are salient studies from this review [60].

Some 44% of TBI patients experience brain hypoxia, which can result in hypoperfusion and severe deficits of physical, psychological, and cognitive functions [61-68]. In a double-blind study of 50 patients suffering chronic cerebral insufficiency, patients were administered either 120 mg/day Ginko Biloba extract (GBE) or placebo for 1 month prior to assessment [69]. Patients who received GBE showed improvements in motor activity, speech comprehension/production, and mood, as well as a reduction in dizziness [69]. Further, in a placebo-controlled, double-blind trial, GBE was administered at a dosage of 160 mg/day for 6 weeks to 60 patients with cerebral insufficiency. Subjects from the GBE group were found to have had progressive improvement in concentration and reduction in fatigue. Between the second and fourth weeks of treatment, two-thirds of the patients on GBE showed improvement compared with one-fifth of the patients on placebo [69].

Moreover, in a double-blind, placebo-controlled study researching the effects of GBE on patients diagnosed with mild to moderate cerebrovascular insufficiency, 40 outpatients receiving 120 mg/day GBE for 12 weeks showed improvement on clinical assessment and on self-rating scales monitoring changes in dizziness, tinnitus, headaches, and hearing loss [70]. Eighteen out of 20 patients showed statistically significant improvements in tinnitus, dizziness, and in the frequency and severity of headaches [71]. Another study, involving 80 patients with cerebrovascular insufficiency, used a double-blind, placebo-controlled, crossover design to test the effects of GBE on perceptual reasoning [72]. Group A received GBE for the first 45 days and placebo for the remainder of the trial, and Group B initially received placebo followed by GBE. Patients in the GBE versus placebo treatment groups displayed significant improvement on the block design subtest of the Wechsler Adult Intelligence Scale [72] and on a visual–spatial construction task. It should be noted, however, that an improvement of 0.7 points on the block design subtest of the Wechsler Adult Intelligence Scale, while being statistically significant, is unlikely to represent a clinically meaningful change [72].

Le Bars and colleagues conducted a 52-week double-blind, randomized placebo-controlled, multicenter clinical trial consisting of 309 patients with AD and multi-infarct dementia to study the efficacy and safety of EGb 761 (24% ginkgo-flavone glycosides and 6% terpenoids [73]). Patients were administered either EGb (120 mg/day) or placebo. Evaluable data were obtained from 202 of the original 309 patients at the 52-week end-point analysis. The cognitive subscale of the AD Assessment Scale, which was used to assess cognitive function in these patients, displayed positive changes in the patients who received EGb. Using the literature-based cutoff score of +4 as an indicator of change, 14% of the people in the placebo group displayed a positive change compared with 27% of the EGb treatment group [74]. The effect size appeared to be independent of age or severity of symptoms at baseline. When clinical symptom severity and treatment response were assessed, however, no differences were noted [74].

Overall, these findings suggest that ginkgo’s broad spectrum of pharmacologic effects allows it to be used in the treatment of various neurologic functions (for example, cognitive, mood, motoric, headache, and motivational) that are associated with both AD and TBI (for example, cerebral insufficiency).

However, difficulties may still remain with brain bioavailability. This difficulty applies not so much with GBE but with other flavonoids, as will be expanded upon [60]. Several studies have been conducted to understand the pharmokinetics and pharmacodynamics of GBE. For example, expired radiolabeled ^i4^C–CO_2_ extract was administered orally in a rat model, and it was found that 16% of the administered dose was excreted in the first 3 hours and a total of 38% after 72 hours. Twenty-one percent of the radiolabeled extract was excreted in the urine and 29% was excreted in the feces [75]. Total absorption reached at least 60%. Regarding the evaluation of blood-specific activity data, the pharmacokinetics of Ginko Biloba follow a two-compartment process. In the first-order phase, the biologic half-life is approximately 4.5 hours. In the second-order phase, the drug was distributed through plasma, followed by a gradual uptake after 48 hours [75]. The upper gastrointestinal tract was also an absorption site, as was neuronal, glandular, and ocular tissue [75].

In a study in which possible or probable AD patients received an oral dose of standardized extract of dry Ginko Biloba leaves, electroencephalogram changes within 3 hours showed that the extract was adequately absorbed, metabolized, and crossed the blood–brain barrier [76]. GBE, or its constituents, has exhibited half-lives ranging from 2 to 4 hours while activity levels peak at 1.5 to 3 hours in animal and human models [60]. Regarding the mechanism of action of this flavonoid, GBE was shown to have activity both centrally and peripherally modulating electrochemical, physiologic, neurologic, and vascular systems in animals and humans with few adverse side effects or drug interactions. As such, GBE may show promise in patients with neurologic sequelae associated with both AD and TBI [60].

Green tea-derived epigallocatechin gallate (EGCG) has anti-amyloidogenic and anti-inflammatory properties in AD mouse models, but the comparable effective dose of EGCG in humans may exceed clinical convenience and/or safety. We previously found that fish oil enhanced bioavailability of EGCG versus EGCG treatment alone (*P* <0.001). Fish oil and EGCG therefore synergistically inhibit cerebral Aβ deposits (*P* <0.001). This finding supports the use of fish oil supplementation with ECGC in order to have significant therapeutic potential for the treatment of AD or TBI [77].

One element of therapeutic animal studies is the type of simulated TBI: focal or diffuse. The majority of studies use a type of mechanical pneumatic or fluid percussion applied to the brain. It is common for both focal and diffuse damage to occur as the result of the same event; so for the purposes of this review we will treat both damage types the same in terms of AD risk. Further the literature does not differentiate diffuse versus focal in terms of AD risk [78]. For example, Di Giovanni and colleagues found that activation of cell cycle proteins after TBI is associated with cell death and caspase activation in neurons, but with proliferation of astrocytes and microglia [79]. This study was conducted over 17 days post injury in male Sprague–Dawley rats. Moreover, cell cycle inhibition by the flavonoid flavopiridol reduced neuron cell death and glial proliferation. Importantly, these changes were paralleled by a significant reduction in lesion volume and by nearly complete functional recovery [79]. In another study, rats were subjected to controlled cortical impact injury and then injected with the flavonoid baicalein (30 mg/kg) or vehicle immediately after injury or daily for 4 days. Improved functional recovery and reduced contusion volumes up to day 28 post injury were observed [80]. These changes were associated with significantly decreased levels, at the contusion site, of TNFα, IL-1β and IL-6 mRNA at 6 hours, and cytokine protein on day 1 post injury – suggesting that the neuroprotective effect of baicalein may be related to a decreased proinflammatory response following the injury [80].

In addition to our work on EGCG in AD mouse models, others found EGCG increased the number of surviving neuronal cells 1, 3, and 7 days post TBI (pneumatic-controlled injury device at 10 weeks of age) and provided an improvement in cerebral dysfunction in 6-week-old male Wistar rats. The authors suggest consumption of water containing EGCG pre and post TBI inhibits free radical-induced neuronal degeneration and apoptotic cell death around the damaged area, resulting in the improvement of cerebral function following TBI [81]. Furthermore, we have found that EGCG promotes nonamyloidogenic processing of APP in mice, resulting in elevations of the neurotrophic soluble APPα [82]. Importantly, soluble APPα reduces neuronal injury and improves functional outcome following diffuse traumatic brain injury in rats [83,84]. In addition we have found that EGCG reduces APC-like microglia and re-polarizes them to phagocytic-like microglia [77,82,85-90].

We and others have also demonstrated that flavonoids significantly suppressed the activation of inflammatory pathways involved in TBI and AD, including NF-κB as well as mitogen-associated protein kinase pathways in activated microglia, resulting in an attenuation of the production of inflammatory molecules [85,91,92]. Luteolin, a flavonoid from celery and green peppers, was recently shown to suppress lipopolysaccharide (LPS)-induced IL-6 protein and mRNA expression by inhibiting activator protein-1 activation and phosphorylation of JNK in the murine microglial cell line BV-2 [91]. IL-6 is among the first cytokines upregulated post TBI [93]. Moreover, when mice were provided drinking water supplemented with luteolin before treatment with LPS, plasma IL-6 and IL-6 mRNA in the hippocampus were reduced compared with those not receiving luteolin [91]. In another study, luteolin affected the microglial transcriptome leading to an anti-inflammatory, anti-oxidative, and neuroprotective phenotype [94]. In further support we found that apigenin and luteolin also suppressed microglia TNFα and IL-6 productions stimulated by IFNγ in the presence of CD40 ligation [95].

In further support of the study by Di Giovanni and colleagues mentioned earlier [79], microglia are the major cells in the brain that generate inflammatory molecules including cytokines, superoxide, and nitric oxide. Previous studies reported that inhibition of these molecules is beneficial for mitigating neurodegenerative disorders [85] including TBI [79]. In the inflammatory response, NF-κB and activator protein-1 are important transcription factors, and mitogen-associated protein kinase (ERK1/2, p38 and JNK) pathways are involved in modulating inflammatory gene expression**.**

Furthermore, studies have shown that flavonoids exert neuroprotection through inhibiting microglia activation and the subsequent release of various inflammatory molecules. For example, pretreatment with luteolin attenuated inflammatory mediators (IL-1β, TNFα, nitric oxide, and prostaglandin E2) produced by LPS-stimulated microglia [96]. Supernatant from LPS-stimulated microglia caused discernible death in N2a cells (a neuroblastoma cell line). However, treating microglia with luteolin prior to LPS reduced neuronal cell death caused by conditioned supernatants [96]. Incubating N2a cells with luteolin did not protect them from supernatants from LPS-stimulated microglia, indicating that luteolin protects neurons by acting exclusively on microglia [96]. Zheng and colleagues also reported that the flavonoid fisetin decreased TNFα and nitric oxide production and significantly suppressed nuclear translocation of NF-κB and phosphorylation of p30 mitogen-associated protein kinase in the LPS-stimulated BV-2 microglia cells [97]. In addition, fisetin reduced cytotoxicity of LPS-stimulated microglia toward B35 neuroblastoma cells in a co-culture system [97].

Inhibition of microglia by wogonin reduced cytotoxicity when co-cultured with pheochromocytoma PC12 cells , supporting a neuroprotective role for wogonin *in vitro*[98]. Other studies have shown that resveratrol, quercetin, or genistein diminished neuronal cell death induced by microglial activation [99,100].

## Conclusions

The inflammatory-based progression of brain injury has been shown to be active in humans for up to 17 years post-TBI. The proinflammatory, APC-like microglial phenotype is a common mechanistic link between both TBI and later development of AD. We and others have shown that naturally occurring flavonoids safely and effectively promote the neuroprotective, anti-inflammatory, phagocytic phenotype. Taken together, these results indicate that flavonoids may be important bioactives for attenuating microglia activation and neuronal cell damage by inflammatory conditions initially initiated by TBI. Ultimately, modulation of microglial phenotype with flavonoids or other compounds provides an avenue for abrogation of secondary cell death and behavioral symptoms that may mediate the progression of TBI and AD.

## Abbreviations

Aβ, amyloid beta; AD, Alzheimer ’s disease; APC, antigen-presenting cell; APP, amyloid precursor protein; EGCG, epigallocatechin gallate GBE, Ginko Biloba extract; IFN, interferon; IL, interleukin; JNK, c-Jun-N-terminal kinase; LPS, lipopolysaccharide; NF, nuclear factor; TBI, traumatic brain injury; Th, T-helper type; TNF, tumor necrosis factor.

## Competing interests

PRS is a founder of NaturaTherapeutics, Inc., and has a consulting relationship but no employment with NaturaTherapeutics, Inc.; CVB is a consultant for NaturaTherapeutics, Inc. NT-020 is a patented formulation which includes flavonoids that is marketed by NaturaTherapeutics, Inc. The patent is held by PRS the University of South Florida and the Veterans Administration.

## Authors’ contributions

BG performed the background search and completed the first draft of the manuscript. DO conducted the literature search and assisted in the initial manuscript preparation. PRS and CVB contributed to the background material regarding which cytokines are promoted by TBI. BG and JT conceived the review subject. RV assisted with the response to the critique. All authors read and approved the final manuscript.
